# *Toxoplasma gondii* Tyrosine-Rich Oocyst Wall Protein: A Closer Look through an In Silico Prism

**DOI:** 10.1155/2021/1315618

**Published:** 2021-10-14

**Authors:** Ali Asghari, Hamidreza Majidiani, Taher Nemati, Mohammad Fatollahzadeh, Morteza Shams, Razi Naserifar, Bahareh Kordi

**Affiliations:** ^1^Department of Medical Parasitology and Mycology, School of Medicine, Shiraz University of Medical Sciences, Shiraz, Iran; ^2^Zoonotic Diseases Research Center, Ilam University of Medical Sciences, Ilam, Iran; ^3^Department of Parasitology and Mycology, School of Medicine, Isfahan University of Medical Sciences, Isfahan, Iran; ^4^Department of Basic Medical Sciences, Neyshabur University of Medical Sciences, Neyshabur, Iran

## Abstract

Toxoplasmosis is a global threat with significant zoonotic concern. The present in silico study was aimed at determination of bioinformatics features and immunogenic epitopes of a tyrosine-rich oocyst wall protein (TrOWP) of *Toxoplasma gondii*. After retrieving the amino acid sequence from UniProt database, several parameters were predicted including antigenicity, allergenicity, solubility and physico-chemical features, signal peptide, transmembrane domain, and posttranslational modifications. Following secondary and tertiary structure prediction, the 3D model was refined, and immunogenic epitopes were forecasted. It was a 25.57 kDa hydrophilic molecule with 236 residues, a signal peptide, and significant antigenicity scores. Moreover, several linear and conformational B-cell epitopes were present. Also, potential mouse and human cytotoxic T-lymphocyte (CTL) and helper T-lymphocyte (HTL) epitopes were predicted in the sequence. The findings of the present in silico study are promising as they render beneficial characteristics of TrOWP to be included in future vaccination experiments.

## 1. Introduction

The model apicomplexan, *Toxoplasma gondii* (*T. gondii*), virtually infects a large number of warm-blooded animal species including humans [1]. Reportedly, one-third of the global population has shown serological traits of a previous exposure to the parasite [2]. Also, the parasite is of veterinary importance, as a well-known abortifacient among domestic livestock [3]. Within feline intestine, gametogony and sporogony occur in order to develop unsporulated oocysts. The latter are shed *via* feces into the environment, become infective, and contaminate food/water supplies [4]. Additionally, fast-replicating tachyzoites (transfusion-mediated and congenital infection) and slow-dividing bradyzoites (cyst-contaminated muscle tissues and organ transplant) are involved in alternative transmission pathways [5]. Notwithstanding its widespread prevalence, *T. gondii* infection rarely results in clinical disease in immunocompetent individuals, whereas a decreased immune status, such as the case in pregnancy and/or immunosuppressive disorders, may pave the way for the opportunistic parasite to vividly invade to the unborn *via* placenta or to central nervous system (CNS) tissues, respectively [6].

Disappointingly, the present chemotherapeutic options for acute infections and/or recrudescence of infection are only active against tachyzoite stages with a number of side-effects reported in treated patients [7]. Thereby, employing preventive measures such as a One-Health vaccination approach more sufficiently benefit the world [8]. Since *Toxoplasma* is an obligatory intracellular organism, T CD_8_^+^ responses with interferon gamma (IFN-*γ*) upsurge are the preferred immunity to combat acute infection. However, humoral responses are also advantageous, in particular following an oral infection with bradyzoites or oocysts [9]. Despite over 30 years of preclinical and clinical *Toxoplasma* vaccination using various platforms, no commercial human vaccine is registered yet [10]. The only available vaccine is the so-called “Toxovax®,” a live attenuated strain (S48) of *T. gondii*, for prevention of abortion in sheep, but it cannot be used in humans for safety issues [11]. The multistage life cycle of *Toxoplasma* requires expression of a large number of proteins during parasite stage-to-stage transition. Surface antigens (SAGs) along with organellar proteins including micronemes (MICs), rhoptries (ROPs), dense granule antigens (GRAs), and their associated molecules have been nominated as the main repository containing valuable vaccine candidates. Interestingly, some of these proteins are stage-specific (SAG1 in tachyzoite), while some may associate with multiple stages (MIC4, MIC13, ROP2, GRA8, GRA14) [12].

Although human *T. gondii* infection acquired *via* tissue cysts could be handled through proper cooking of meat products, but oocysts can thoroughly contaminate water streams, soils, and foodstuffs and survive for a long time in moist surrounding as well as fresh/marine waters [13]. This outstanding ability of oocysts is not yet fully understood, but the underlying mechanism may lie within key molecular components in the oocyst walls [14, 15]. In light of advances in omics-based technologies, an increasing trend has been raised to precisely decipher the parasite biology and to characterize critical immunocorrelates of *T. gondii* protection [16]. In this sense, bioinformatics methods have more facilitated the identification of biophysical features and novel immunogenic antigens/peptides in different *Toxoplasma* infectious stages [17]. The present in silico study was performed to have a closer look at bioinformatics properties of the *T. gondii* tyrosine-rich oocyst wall protein (TrOWP) and its immunodominant cytotoxic T-lymphocyte (CTL) and helper T-lymphocyte (HTL) as well as B-cell epitopes using comprehensive immunoinformatics tools.

## 2. Methods

### 2.1. Amino Acid Sequence Retrieval

The complete amino acid sequence of *Toxoplasma* TrOWP was gathered as FASTA format *via* the Universal Protein Resource (UniProt), available at https://www.uniprot.org/, under entry number of V5LE41 for further bioinformatics analyses.

### 2.2. Prediction of Antigenicity, Allergenicity, Solubility, and Physico-Chemical Properties

Antigenicity forecasting of the protein was done through two network-based tools: VaxiJen v2.0 (http://www.ddg-pharmfac.net/vaxijen/VaxiJen/VaxiJen.html) and ANTIGENpro (http://scratch.proteomics.ics.uci.edu/). VaxiJen v2.0 is a free web tool that predicts protein antigenicity in target organisms (parasite, fungal, virus, bacteria, tumor) with desirable threshold. The prediction is based on physico-chemical features and sequence transformation into uniform vectors of amino acid characteristics *via* auto cross covariance (ACC) [18]. As well, particular microarray data is used by ANTIGENpro online tool, in a pathogen- and alignment-free manner [19]. Allergenic feature of the protein was evaluated using AllerTOP v2.0 (https://www.ddg-pharmfac.net/AllerTOP/) and AlgPred (http://crdd.osdd.net/raghava/algpred/) servers. AllerTOP utilizes E-descriptors, k-nearest neighbors, and auto/cross variance transformation algorithms [20]. Also, AlgPred employs several machine learning algorithms to determine IgE-specific epitopes and MEME (Multiple Em for Motif Elicitation)/MAST (Motif Alignment and Search Tool) allergen motifs [21]. Protein solubility was calculated using two web servers: SOLpro (http://scratch.proteomics.ics.uci.edu/) and Protein-Sol (https://protein-sol.manchester.ac.uk/). SOLpro employs a two-stage support vector machine (SVM) algorithm to check the solubility upon overexpression in *Escherichia coli* (*E. coli*) [22]. Based on the population average of the experimental dataset in Protein-Sol server, that is 0.45, any solubility value above this score is predicted to be highly soluble [23]. Finally, the physico-chemical functions of the TrOWP were determined using ExPASy ProtParam web server (https://web.expasy.org/protparam/), showing the protein molecular weight (MW), positively and negatively charged residues, isoelectric point (pI), *in vitro* and *in vivo* estimated half-life, instability index, aliphatic index, and grand average of hydropathicity (GRAVY) [24].

Prediction of Transmembrane Domain, Signal Peptide, and Posttranslational Modification (PTM) Sites

The presence of putative transmembrane domains was evaluated using TMHMM online server, available at http://www.cbs.dtu.dk/services/TMHMM/. Moreover, the prediction of PTM sites in the protein sequence such as N-glycosylation, palmitoylation, phosphorylation, and acetylation was predicted via NetNGlyc 1.0 (http://www.cbs.dtu.dk/services/NetNGlyc/) [25], CSS Palm (http://csspalm.biocuckoo.org/) [26], NetPhos 3.1 (http://www.cbs.dtu.dk/services/NetPhos/) [27], and GPS-PAIL 2.0 (http://pail.biocuckoo.org/links.php) [28] web servers, respectively. The prediction settings were default, except for NetNGlyc and acetylation, which was based on “all Asn residues” and “all types,” correspondingly. Also, SignalP-5.0 server, available at http://www.cbs.dtu.dk/services/SignalP/, was aimed at prediction of putative signal peptide in the protein sequence [29].

### 2.3. Prediction of Secondary and Tertiary Structures

Two web servers were used for secondary structure analysis, including Garnier–Osguthorpe Robson (GOR) IV server with 64.4% mean accuracy (https://npsa-prabi.ibcp.fr/cgi-bin/npsa_automat.pl?page=/NPSA/npsa_gor4.html) [30] and position specific iterated prediction (PSIPRED) on PSI-BLAST outputs for similarity determination (http://bioinf.cs.ucl.ac.uk/psipred/) [31]. Further structural analysis was aimed at predicting the three-dimensional (3D) aspect of the TrOWP protein. Hence, the protein sequence was submitted to the I-TASSER web server for homology modelling (https://zhanglab.ccmb.med.umich.edu/I-TASSER/) [32]. “I-TASSER (Iterative Treading ASSEmbly Refinement) is a best-ranked server which is used to create automated protein structures and prediction. Upon submission of an amino acid sequence, I-TASSER works to design a 3D atomic model by utilizing the multiple threading alignments and iterative structural assembly simulations” [33, 34].

### 2.4. Refinement of the 3D Structure and Validations

The best-fit, high-ranked 3D model provided by I-TASSER server was subsequently subjected for structural rehashing and relaxation purposes, using GalaxyRefine server (http://galaxy.seoklab.org/cgi-bin/submit.cgi?type=REFINE). This server is one of the best refining tools, which enhances the predicted structure through formation of side chains and their repacking, thereby providing an overall relaxation in the structure *via* dynamic simulations. Five refined models are yielded following calculations, based on various qualifying scores including global distance test-high accuracy (GDT-HA), root mean square deviation (RMSD), MolProbity, Clash score, Poor rotamers, and Rama favored [35]. In the next step, the quality of the refining process was checked through Ramachandran plot analysis of Zlab (https://zlab.umassmed.edu/bu/rama/) and ERRAT tool of SAVES 6.0 server (https://saves.mbi.ucla.edu/). “ERRAT analyzes the statistics of non-bonded interactions among various atom types by comparison with statistics from highly-refined structures” [36, 37]. Furthermore, Ramachandran analysis is directed towards protein structure confirmation through energetically allowed and disallowed dihedral angles of psi (*ψ*) and phi (*ϕ*) per amino acid [38, 39].

### 2.5. Prediction of Continuous and Conformational B-Cell Epitopes

Identification of B-cell epitopes is a necessary step in vaccine design. For this purpose, a multistep process was designed to cover shared epitopes found through several web servers [40]; hence, three servers were utilized including BCPREDS (http://ailab-projects1.ist.psu.edu:8080/bcpred/), ABCpred (http://crdd.osdd.net/raghava/abcpred/) [41], and SVMTriP (http://sysbio.unl.edu/SVMTriP/) [42]. BCPREDS server provides user-friendly fixed-length (BCPred and AAP) and flexible-length (FBCPred) epitope prediction modes, based on the desired threshold using subsequent kernel (SSK) and SVM algorithms [43]. Here, a fixed-length prediction (14 amino acids) with 75% threshold was chosen. Cross-validation of predicted epitopes was done by other two servers. Ultimately, shared linear B-cell epitopes were further assessed in terms of antigenicity, allergenicity, and water solubility through VaxiJen v2.0, AllerTOP v2.0, and PepCalc (https://pepcalc.com/) online servers, respectively. As well, the protein sequence was subjected to Bcepred server in order to forecast continuous B-cell epitopes according to physico-chemical properties such as hydrophilicity, flexibility, accessibility, turns, exposed surface, polarity, and antigenic propensity (http://crdd.osdd.net/raghava/bcepred/). Additionally, for prediction of conformational B-cell epitopes, ElliPro tool of IEDB database was ran using default settings of 0.5 min score 6 Å max distance, which performs by neighbor residue clustering, residue protrusion index (PI), and protein shape appraisal [44].

### 2.6. Prediction of Mouse and Human CTL/HTL Epitopes

Those epitopes with specific binding capacity to mouse major histocompatibility complex- (MHC-) I and II molecules were predicted against MHC-I alleles (H2-Db, H2-Dd, H2-Kb, H2-Kd, H2-Kk, and H2-Ld) and MHC-II alleles (H2-IAb, H2-IAd, and H2-IEd), *via* IEDB MHC-I binding (http://tools.iedb.org/mhci/) and MHC-II binding (http://tools.iedb.org/mhcii/) tools, respectively. Epitope prediction was done using IEDB recommended 2020.09 (NetMHCPan EL 4.1) method for MHC-I (CTL) epitopes and IEDB recommended 2.22 for MHC-II (HTL) peptides. Each epitope is assigned a percentile rank, which inversely associate with the affinity, so that the lower is the percentile rank; the higher is the epitope affinity to the respective MHC molecule. In the following, mouse MHC-I binding epitopes were screened regarding immunogenicity, allergenicity, and hydrophobicity via IEDB MHC-I immunogenicity tool (http://tools.iedb.org/immunogenicity/), AllerTOP v2.0, and Peptide2 (https://peptide2.com/N_peptide_hydrophobicity_hydrophilicity.php) online tools, respectively. Also, mouse MHC-II binding epitopes were further evaluated by antigenicity, allergenicity, IFN-*γ*, and IL-4 induction using VaxiJen v2.0, AllerTOP v2.0, IFNepitope (http://crdd.osdd.net/raghava/ifnepitope/), and IL4pred (http://crdd.osdd.net/raghava/il4pred/) servers, correspondingly.

Given human CTL epitopes, NetCTL 1.2 web server was used, which predicts CTL epitopes with respect to 12 major MHC supertypes (threshold 0.75). In order to cover up to 90% of human leukocyte antigen (HLA) in the global population, we utilized top five frequent supertypes (A1, A2, A3, A24, and B7) for CTL epitope prediction [45]. Similar to mouse MHC-I epitopes, screening was performed regarding immunogenicity, allergenicity, and hydrophobicity. Moreover, human HTL epitopes were predicted using IEDB MHC-II tool with respect to full-HLA reference set, along with antigenicity, allergenicity, IFN-*γ*, and IL-4 induction analyses.

## 3. Results

### 3.1. General Characteristics of *T. gondii* TrOWP

The outputs of VaxiJen v2.0 and ANTIGENpro servers demonstrated that the protein possesses antigenic feature with 0.8769 (threshold 0.5) and 0.879297 scores, respectively. Moreover, AllerTOP v2.0 and AlgPred predictions revealed no allergenic traits, no IgE specific epitopes, and no MEME/MAST motifs in the sequence. Protein solubility results using SOLpro and Protein-Sol servers showed that TrOWP is highly soluble with 0.623441 and 0.810 scores, respectively. Based on ProtParam server outputs, the protein contained 236 amino acid residues, molecular weight of 25579.15, and a relatively acidic speculated pI of 4.85. There were more negatively charged (Asp + Glu) residues in the sequence (38), than positively charged residues (Arg + Lys) (27). Estimated half-life was calculated to be 30 h (mammalian reticulocytes, *in vitro*), >20 h (yeast, *in vivo*), and >10 h (*E. coli*, *in vivo*). Also, the protein was stable (instability index: 29.51), moderately thermotolerant (54.24), and hydrophilic nature (GRAVY score: -0.758).

### 3.2. Transmembrane Domain, Signal Peptide, and PTM Sites of TrOWP

The output of TMHMM server determined no transmembrane domains in the protein sequence. Of note, there were several PTM sites in the sequence. NetPhos 3.1 server predicted 26 phosphorylation sites (14 serine, 9 threonine, and 3 tyrosine), while 13 acetylation sites were present in the sequence, according to GPS-PAIL tool. However, there was no N-glycosylation and palmitoylation sites in TrOWP. SignalP-5.0 server revealed the presence of a standard signal peptide (Sec/SPI) in the sequence with a 0.9967 likelihood.

### 3.3. Secondary and Tertiary Structure Prediction

**Figure 1:**
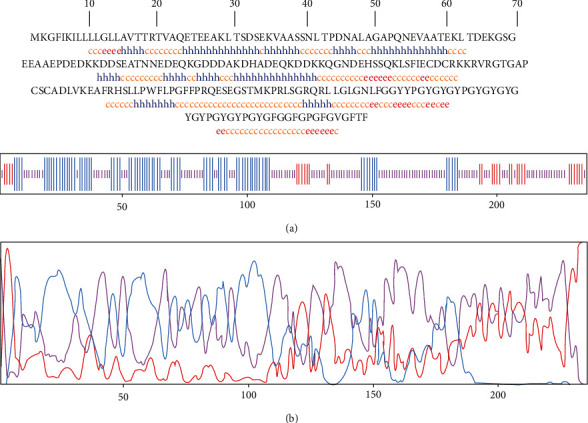
Secondary structure prediction for TrOWP using GOR IV web server. (a) Sequence-based prediction results indicated that 54.24%, 33.05%, and 12.71% of the sequence are dedicated to the random coil (yellow C), alpha helix (blue H), and extended strand (red E), respectively. (b) Graphical illustration of the secondary structure by GOR IV server.

**Figure 2:**
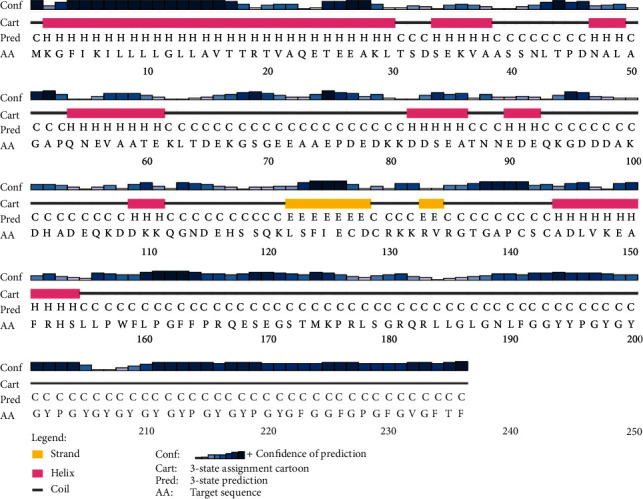
Graphical illustration of secondary structure prediction of TrOWP using PSIPRED server.

Based on GOR IV and PSIPRED output, three secondary structure constituents were present in the sequence, including 128 (54.24%) random coil and 78 (33.05%) alpha helix as well as 30 (12.71%) extended strand. The graphical representation of secondary structure prediction by GORV and PSIPRED servers is illustrated in Figures 1 and 2, respectively.

**Figure 3:**
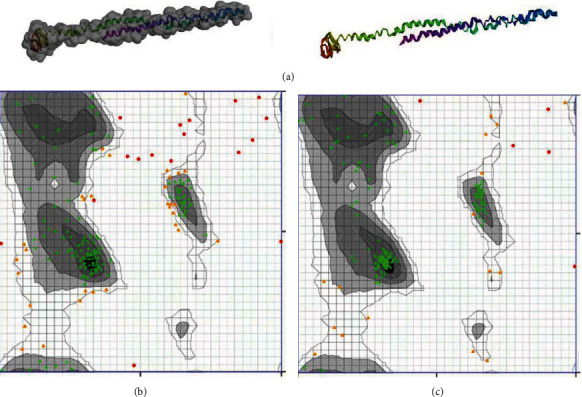
Protein 3D modelling and validation. (a) The final tertiary model of the TrOWP gathered following homology modelling by I-TASSER web server, as shown in ribbon and surface. (b) Ramachandran plot analysis of the initial model showed 74.33%, 16.57%, and 9.09% of the residues in favored, allowed, and outlier areas, respectively. (c) After refinement, 88.77%, 8.56%, and 2.67% of the residues are in favored, allowed, and outlier regions, respectively.

Subsequent homology modelling analysis by I-TASSER server predicted five tertiary models based on top 10 threading templates using LOMETS threading approach. Each predicted model is appointed a C-score as confidence index, ranging between -5 and 2, where higher C-score values indicate a higher confidence in prediction. Provided models using I-TASSER had a C-score from -4.3 to -5. Model number 1 with C-score -4.3, estimated TM-score 0.26 ± 0.08 and estimated RMSD of 16.3 ± 3.0 Å, was chosen for further refining and validations (Figure 3(a)).

### 3.4. Tertiary Structure Refinement and Validations

GalaxyRefine server output was shown as five refined models. Based on qualifying parameters of GDTHA (0.8951), RMSD (0.579), MolProbity (2.789), Clash score (34.5), Poor rotamers (1.1), and Rama favored (79.5), model number 2 was the best refined model. Subsequently, two web servers validated the refining process, including Zlab Ramachandran analysis and ERRAT. The overall quality factor of ERRAT in crude model was 71.491, which was improved to 74.561 after refinement. Moreover, Ramachandran plot analysis demonstrated that 74.33%, 16.57%, and 9.09% of residues are located in favored, allowed, and outlier regions, respectively. Following refinement, these were improved to 88.77%, 8.56%, and 2.67% in favored, allowed, and outlier areas, correspondingly (Figures 3(b) and 3(c)).

Prediction of Linear and Conformational B-Cell Epitopes

**Figure 4:**
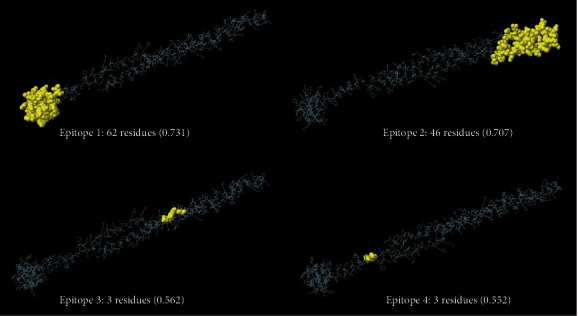
Predicted conformational B-cell epitopes of TrOWP by ElliPro tool of IEDB analysis resource. Length and score of each protein are provided.

Multistep linear B-cell epitope prediction revealed nine shared peptides among three web servers (BCPREDS, ABCpred, and SVMTriP) with high antigenicity score and good water solubility and without allergenic properties (Table 1 and Supplementary File 1). Also, the linear B-cell epitopes predicted on the basis of physico-chemical parameters are tabulated in Table 2. In addition, ElliPro tool of the IEDB server demonstrated four conformation epitopes in the protein sequence, with length and scores as follows: (i) 62 residues (0.731), (ii) 46 residues (0.707), (iii) 3 residues (0.562), and (iv) 3 residues (0.552) (Figure 4).

Prediction of Mouse and Human CTL/HTL Epitopes

Mouse MHC-I epitopes were predicted with respect to six MHC-I alleles (H2-Db, H2-Dd, H2-Kb, H2-Kd, H2-Kk, and H2-Ld) with subsequent immunogenicity, allergenicity, and hydrophobicity analyses (Table 3). Also, mouse MHC-II binding peptides were predicted against three MHC-II alleles (H2-IAb, H2-IAd, and H2-IEd) with subsequent screenings (Table 4). Specific human CTL and HTL epitopes are, also, provided in Tables 5 and 6, respectively.

## 4. Discussion

One-third of the global population is affected by the apicomplexan protist, *T. gondii*, and its clinical significance is conspicuous in pregnant women and immunocompromised patients [46, 47]. Despite over decades of research in the field of toxoplasmosis vaccination using various antigens and immunization platforms, a human vaccine is still lacking [10]. In the molecular era, the combination of mathematical algorithms with outstanding large depository of biological data has yielded the interdisciplinary bioinformatics science. “Immunoinformatics” is a subdivision aimed at characterization of novel vaccine candidates, their biophysical features, immunogenic epitopes, and evaluation of intermolecular interactions as well as the dynamics of host responses to injected vaccine through in silico approaches. Some advantages are anticipated using this novel approach, including (i) time- and cost-effectiveness; (ii) precisely targeted, durable immune response with desired polarity in cellular components; and (iii) abolition of unfavorable responses through specific, epitope-based construct design [48]. The present *in silico* study was done to identify the bioinformatics features of the poorly known *Toxoplasma* TrOWP, along with identification of potential CTL, HTL, and B-cell epitopes for future vaccine design.

*Toxoplasma* oocysts are shed *via* felid's feces as unsporulated form, where under optimum climatic conditions (20-25°C, moist soil) turn into infective sporulated oocysts [49]. Early studies demonstrated that *T. gondii* infection was lacking in cat-free islands, emphasizing the significance of oocysts in transmission dynamics and infection maintenance [50, 51]. Such infective stages are highly resistant to a wide range of temperatures (-20°C to +37°C) [52], salinity up to 15 ppt (parts per thousand) [53] and chemical inactivation agents used in water treatment supplies [54], whereas they succumb to temperatures over 45°C as well as desiccation [55–57]. Therefore, oocysts ring the alarm of a possible health hazard in regions where drinking water treatment plants are only based on chemical disinfection without further filtration [54]. Insights into structural conformation of oocyst wall components would represent us the key for such a large extent of environmental resistance by oocysts [14, 15]. This intuition was mostly gained from pioneering investigations on the oocyst walls of other apicomplexan members, including *Cryptosporidium parvum* and *Eimeria* species [13]. Proteomics analyses during the last decade led to the identification of approximately 225 proteins in fractions of *T. gondii* oocyst wall, with the predominance of PAN-domain containing, cysteine or tyrosine rich proteins [15, 58, 59]. The latter were initially discovered in *Eimeria maxima* within macrogametes type-2 wall forming bodies and inner layer of oocyst wall, showing high rate of conservation across *Eimeria* species [60–62]. Detailed transcriptomic and proteomic information suggested that such proteins are different in *T. gondii*, localizing in both layers of oocyst walls [59, 63]. Here, a TrOWP of *T. gondii* was further characterized using a set of bioinformatics web servers.

The basic features of a good vaccine candidate are antigenicity and lack of allergenicity; accordingly, TrOWP was showed to have adequate antigenic scores *via* analysis by VaxiJen v2.0 (0.8769) and ANTIGENpro (0.879297) web servers. It was, also, considered as a nonallergen protein by AllerTOP v2.0 server, without IgE specific epitopes and MEME/MST motifs, as demonstrated by AlgPred server. The ProtParam server revealed some of the critical physico-chemical parameters of the protein, so that this 236 amino acid proteins possessed a MW of 25.57 kDa, suggesting adequate immunogenicity value, since molecules over 5-10 kDa are strong immunogens [64]. The pI is defined as the pH at which net charge turns zero, which for this protein was relatively acidic in nature (4.85). Negatively charged residues (Asp + Glu) were prevalent in the sequence than positively charged residues (Arg + Lys). According to instability index of 29.51 and aliphatic index of 54.24, TrOWP was a stable and moderately thermotolerant molecule. In fact, higher aliphatic index demonstrates higher tolerability to vast range of temperatures [65]. Moreover, the protein was highly hydrophilic in nature, as evidenced by a negative GRAVY score (-0.785). Protein solubility is another important factor in purification experiments. Two web servers, SOLpro a d Protein-Sol, substantiated that TrOWP is a highly soluble molecule with 0.623441 and 0.810 scores, respectively. Totally, such biophysical characteristics are fundamental for further extraction/purification purposes in the molecular laboratory experiments.

Synthesized proteins may be directed towards cellular secretory pathway for several purposes such as excretory/secretory antigen, virulence factor, and/or structural molecule; hence, they are highlighted by a signal peptide [66]. In the present in silico study, SignalP-5.0 web tool assisted us for detecting three types of signal peptide: “standard secretory signal peptides transported by the Sec translocon and cleaved by Signal Peptidase I (Sec/SPI), lipoprotein signal peptides transported by the Sec translocon and cleaved by Signal Peptidase II (Sec/SPII), and Tat signal peptides transported by the Tat translocon and cleaved by Signal Peptidase I (TAT/SPI)” [29]. TrOWP only possessed a standard signal peptide with the probability of 0.9967. Crude synthesized proteins undergo several enzymatic modifications, including glycosylation, acetylation, palmitoylation, and phosphorylation, totally known as PTMs [67]. Each modification has its own function, such as protein half-life alteration (glycosylation), signal transduction (phosphorylation), membrane anchoring (acetylation), and enhanced protein hydrophobicity (palmitoylation) [68]. Among PTMs examined in the present study, 26 phosphorylation sites and 13 acetylation sites were only predicted in TrOWP, whereas palmitoylation and N-glycosylation were lacking. In general, prediction of PTMs, in particular for eukaryotic proteins, is beneficial for choosing appropriate expression hosts for recombinant protein production purposes. Complex protein structures can be produced efficiently using yeast- and mammal-based expression machineries [68]. It is believed that “the presence of hydrogen bonds in a polypeptide chain between amino hydrogen and carboxyl oxygen represents the secondary structure, with frequent *α*-helices and *β*-structures” [69]. Moreover, tertiary or 3D structure of a protein is defined by the involved bonds and their interactions. High hydrogen-bond energy among *α*-helices and *β*-structures strongly maintains the protein conformation and, therefore, strengthens the possible interaction with antibodies [70]. Secondary structure prediction using two web servers, GOR IV and PSIPRED, determined the predominance of random coil (54.24%), followed by alpha helix (33.05%) and extended strand (12.71%). In the next step, I-TASSER tool of the Zhang Lab automatically generated high-quality 3D models using TrOWP amino acid sequence. “To select the final models, I-TASSER uses SPICKER program to cluster all the decoys based on the pair-wise structure similarity, and report up to five models which corresponds to the five largest structure clusters and a C-score of higher value signifies a model with a high confidence” [71]. Here, model number 1 was the best-fit model, based on the C-score of -4.3, estimated TM-score of 0.26 ± 0.08, and estimated RMSD of 16.3 ± 3.0 Å. Subsequently, GalaxyRefine server was used to establishment and rehashing side chains, in order to improve the global quality of the structure. The outputs of this server for the selected model were including GDTHA (0.8951), RMSD (0.579), MolProbity (2.789), Clash score (34.5), Poor rotamers (1.1), and Rama favored (79.5). ERRAT and Ramachandran analyses, also, confirmed the quality of the refined model in comparison with the initial model.

Upon oocyst challenge, the gut mucosal barriers are the first place that host body senses the parasite through innate lymphoid cell (ILC) types 1 and 3 [72]. Hence, ILC1 secretes IFN-*γ* and tissue necrosis factor alpha (TNF-*α*), while ILC3 activates CD_4_^+^-dependent T-cell responses in lamina propria. Consequently, sensing the infection through the so-called toll-like receptors (TRLs) would summon macrophages and dendritic cells (DCs) to the infection site, where they produce a Th1-type cytokine, IL-12, to more elicit T CD_4_^+^ and T CD_8_^+^ cells. Furthermore, natural killer (NK) cells are stimulated to secrete another Th1-type cytokine, IFN-*γ*, which is totally required to limit the proliferation of parasites. Additionally, a Th-2 response and IL-4 production is a vital marker for B-cell propagation and differentiation as specific humoral response. On [9] this bases, specific CTL (CD8^+^), HTL (CD4^+^) and linear B-cell epitopes of TrOWP were predicted using various web servers. Regarding linear B-cell epitopes, a cross-checking approach was performed using BCPREDS, ABCpred, and SVMTriP servers to find common epitopic regions in the protein sequence. The final epitopes were further screened with respect to antigenicity, allergenicity, and water solubility. Therefore, the following six peptides were qualified as the best linear B-cell epitopes: “EEAAEPDE,” “TNNEDEQ,” “EPDEDKKDDS,” “QGNDEHSSQ,” “AGAPQNEVAAT,” and “SQKLSFIECDCRK.” Human CTL epitopes were predicted in context of the most frequent MHC-I supertypes (A1, A2, A3, A24, and B7) to cover large number of global population. As well, mouse CTL epitopes were predicted with respect to six mouse MHC-I alleles (H2-Db, H2-Dd, H2-Kb, H2-Kd, H2-Kk, and H2-Ld). All of these peptides were screened regarding immunogenicity, allergenicity, and hydrophobicity. Among human CTL epitopes, “LPWFLPGFF” (B7 supertype) and “FLPGFFPRQ” (A2 supertype) possessed high immunogenicity and hydrophobicity indices, without allergenic traits. Regarding mouse CTL peptides, “RHSLLPWFLPGF” along with “ALAGAPQNEVAA” qualified as best-ranked epitopes. Moreover, prediction of human HTL epitopes was done with respect to HLA reference set of IEDB; accordingly, “HSLLPWFLPGFFPRQ” and “RHSLLPWFLPGFFPR” were two antigenic, nonallergenic HTL peptides (VaxiJen score: 1.2901 vs. 1.1923) with potent capacity for IFN-*γ* induction. Also, “KLSFIECDCRKKRVR” was a highly antigenic MHC-II binding epitope with probable potential for IL-4 induction. With respect to mouse HTL epitopes, “CDCRKKRVRGTGAPC” was a highly antigenic (VaxiJen score: 1.7682), nonallergen IFN-*γ* and IL-4 inducer. As well, “RKKRVRGTGAPCSCA” and “SFIECDCRKKRVRGT” (VaxiJen score: 2.1439 vs. 1.8125) were two mouse MHC-II binding epitopes, which could efficiently induce IL-4 cytokine.

## 5. Conclusion

In spite of over three decades of research in the field of vaccination against toxoplasmosis, finding the most efficient antigen(s) and immunization platform is still a global health challenge. Computer-aided tools assist us to identify immunostimulant regions of a given antigen and better design multiepitope-based vaccine candidates for improving immunization. Previously, several surface and/or excretory/secretory antigens of *T. gondii* were evaluated using bioinformatics tools, while lack of studies on oocyst wall proteins leads us to select *T. gondii* TrOWP. The present study investigated the bioinformatics properties of TrOWP and its potent immunogenic mouse and human CTL/HTL epitopes to show its potential as a vaccine candidate. It was substantiated that TrOWP was a highly antigenic, nonallergenic, and soluble protein with several B-cell-specific and MHC-binding peptides in mouse and human, which highlights its significance for future vaccinology studies to prevent *Toxoplasma* infection as alone or multiepitope formulations in experimental models.

## Figures and Tables

**Table 1:** The final screening of shared linear B-cell epitopes from *T. gondii* tyrosine-rich oocyst wall protein.

B-cell epitopes	Antigenicity	Allergenicity	Solubility
YPGYGYGYPGYGYG	0.2095	No	Poor
EEAAEPDE	0.7107	No	Good
VAASSNLTPDNA	0.5596	Yes	Poor
EPDEDKKDDS	1.4261	No	Good
QGNDEHSSQ	1.7231	No	Good
TNNEDEQ	1.8297	No	Good
AGAPQNEVAAT	1.1823	No	Good
PRLSGRQRLLGL	0.2650	No	Good
SQKLSFIECDCRK	0.8220	No	Good

**Table 2:** Specific B-cell linear epitopes of *T. gondii* tyrosine-rich oocyst wall protein based on different physico-chemical parameters predicted by the Bcepred web server.

Physico-chemical parameter	B-cell epitopes
Hydrophilicity	VAQETEEAKLTSDSEKVA, AGAPQNE, ECDCRKKR, PRQESEGSTMK
Flexibility	EEAKLTSDSE, EKLTDEKGSG, IECDCRKKRVRG, GFFPRQESEGSTMKPRLSGRQ
Accessibility	TRTVAQETEEAKLTSDSEKV, SNLTPDN, ECDCRKKRVRGTG, KEAFRHS, FFPRQESEGSTMKPRLSGRQR
Turns	EATNNEDE, GNDEHSSQ
Exposed surface	QETEEAK, EKLTDEK, ECDCRKKRVRGT
Polarity	VAQETEEAKLTS, IECDCRKKRVRGTG, VKEAFRHSL, FPRQESEGS, KPRLSGRQR
Antigenic propensity	GFIKILLLLGLL, KLSFIECDCR, FRHSLLPWFLP

**Table 3:** Mouse specific MHC-I epitopes predicted by CTLpred server with subsequent screening for immunogenicity, allergenicity, and hydrophobicity.

Mouse MHC-I alleles	T-cell peptide	Immunogenicity score	Allergenicity	Hydrophobicity (%)
H2-Db (MHC-I)	AASSNLTPDNAL	-0.17199	Yes	50
	ALAGAPQNEVAA	0.10695	No	66.67
	FFPRQESEGSTM	-0.11546	No	33.33
H2-Dd (MHC-I)	FGGFGPGFGVGF	0.38454	No	50
	KGFIKILLLLGL	0.06992	Yes	66.67
	RHSLLPWFLPGF	0.24324	No	66.67
H2-Kb (MHC-I)	KGFIKILLLLGL	0.06992	Yes	66.67
	RHSLLPWFLPGF	0.24324	No	66.67
	FGGFGPGFGVGF	0.38454	No	50
H2-Kd (MHC-I)	FFPRQESEGSTM	-0.11546	No	33.33
	GYPGYGYPGYGF	0.09408	No	25
	GFGPGFGVGFTF	0.37342	No	50
H2-Kk (MHC-I)	QESEGSTMKPRL	-0.39637	Yes	25
	EEAKLTSDSEKV	-0.51403	Yes	25
	DLVKEAFRHSLL	-0.01925	Yes	50
H2-Ld (MHC-I)	HSLLPWFLPGFF	0.3799	No	75
	YPGYGYGYGYGY	0.11446	No	8.33
	EAFRHSLLPWFL	0.18378	No	66.67

**Table 4:** Mouse specific MHC-II epitopes predicted by IEDB server with subsequent screening for antigenicity and allergenicity as well as IFN-*γ* and IL-4 induction.

Mouse MHC-II alleles	T-cell peptide	Antigenicity	Allergenicity	IFN-*γ* induction		IL-4 induction	
				Result	Score	Result	Score
H2-IAb (MHC-II)	PDNALAGAPQNEVAA	1.2048	Yes	Negative	-0.6321	Negative	-1.02
	TPDNALAGAPQNEVA	0.9905	No	Negative	-0.6006	Negative	-1.01
	RKKRVRGTGAPCSCA	2.1439	No	Negative	-0.249	Positive	0.39
H2-IAd (MHC-II)	IKILLLLGLLAVTTR	0.6131	No	Negative	4	Negative	-0.75
	LTSDSEKVAASSNLT	1.2417	Yes	Negative	2	Negative	-0.38
	SDSEKVAASSNLTPD	1.0754	No	Negative	2	Negative	-0.41
H2-IEd (MHC-II)	SFIECDCRKKRVRGT	1.8125	No	Negative	-0.3691	Positive	0.29
	CDCRKKRVRGTGAPC	1.7682	No	Positive	1	Positive	0.29
	ADLVKEAFRHSLLPW	0.7519	Yes	Positive	1	Positive	0.23

**Table 5:** Human specific MHC-I epitopes predicted by CTLpred server with subsequent screening for immunogenicity, allergenicity, and hydrophobicity.

Human MHC class I supertype	CTL epitopes	Immunogenicity score	Allergenicity	Hydrophobicity (%)
A1 supertype	GLGNLFGGY	0.15229	No	33.33
	CADLVKEAF	-0.0594	No	55.56
	ATEKLTDEK	-0.08154	Yes	22.22
A2 supertype	LLLLGLLAV	0.0213	No	88.89
	KLTSDSEKV	-0.3295	Yes	22.22
	LLAVTTRTV	0.19494	Yes	55.56
	GLLAVTTRT	0.17551	No	44.44
	FLPGFFPRQ	0.27558	No	66.67
A3 supertype	TMKPRLSGR	-0.16102	No	33.33
	GLGNLFGGY	0.15229	No	33.33
A24 supertype	GYGYPGYGF	0.04506	No	22.22
	GFIKILLLL	-0.07048	No	77.78
	YYPGYGYGY	0.07548	No	11.11
	RLLGLGNLF	0.03966	Yes	55.56
	RHSLLPWFL	0.16924	No	66.67
B7 supertype	YPGYGFGGF	0.19888	No	33.33
	LPWFLPGFF	0.26546	No	88.89
	KPRLSGRQR	-0.14756	No	22.22
	RVRGTGAPC	0.14714	Yes	33.33
	APQNEVAAT	0.14813	No	55.56
	APCSCADLV	-0.1874	No	55.56
	SGRQRLLGL	-0.04936	No	33.33

**Table 6:** Human specific MHC-II epitopes predicted by IEDB server using HLA reference set with subsequent screening for antigenicity and allergenicity as well as IFN-*γ* and IL-4 induction.

Human MHC-II allele	HTL epitope	Method	Percentile rank	Antigenicity	Allergenicity	IFN-*γ* induction		IL-4 induction	
						Result	Score	Result	Score
HLA-DRB1^∗^01 : 01	FIKILLLLGLLAVTT	Consensus (comb.lib./smm/nn)	0.10	0.8754	No	Negative	61	Negative	-0.74
HLA-DRB1^∗^15 : 01	FIKILLLLGLLAVTT	Consensus (smm/nn/sturniolo)	0.93	0.8754	No	Negative	61	Negative	-0.74
HLA-DPA1^∗^01 : 03/DPB1^∗^04 : 01	LVKEAFRHSLLPWFL	NetMHCIIpan	0.95	0.8487	Yes	Negative	-0.0405	Positive	0.02
HLA-DPA1^∗^02 : 01/DPB1^∗^05 : 01	MKGFIKILLLLGLLA	Consensus (comb.lib./smm/nn)	1.10	0.9498	No	Negative	71	Negative	-0.74
HLA-DPA1^∗^01 : 03/DPB1^∗^02 : 01	HSLLPWFLPGFFPRQ	Consensus (comb.lib./smm/nn)	1.40	1.2901	No	Positive	1	Negative	0.15
HLA-DPA1^∗^01 : 03/DPB1^∗^04 : 01	KEAFRHSLLPWFLPG	NetMHCIIpan	1.50	0.7021	Yes	Positive	0.0844	Negative	0.07
HLA-DPA1^∗^03 : 01/DPB1^∗^04 : 02	GFIKILLLLGLLAVT	Consensus (comb.lib./smm/nn)	1.60	0.4212	No	Negative	71	Negative	-0.74
HLA-DPA1^∗^01 : 03/DPB1^∗^04 : 01	HSLLPWFLPGFFPRQ	NetMHCIIpan	1.60	1.2901	No	Positive	1	Negative	0.15
HLA-DPA1^∗^03 : 01/DPB1^∗^04 : 02	MKGFIKILLLLGLLA	Consensus (comb.lib./smm/nn)	1.60	0.9498	No	Negative	1	Negative	-0.74
HLA-DQA1^∗^01 : 01/DQB1^∗^05 : 01	FRHSLLPWFLPGFFP	Consensus (comb.lib./smm/nn)	1.90	1.5483	No	Negative	-0.0952	Negative	0.14
HLA-DPA1^∗^01 : 03/DPB1^∗^04 : 01	EAFRHSLLPWFLPGF	NetMHCIIpan	2.00	0.9177	Yes	Negative	-0.0048	Negative	0.08
HLA-DQA1^∗^01 : 01/DQB1^∗^05 : 01	HSLLPWFLPGFFPRQ	Consensus (comb.lib./smm/nn)	2.00	1.2901	No	Positive	1	Negative	0.15
HLA-DPA1^∗^02 : 01/DPB1^∗^01 : 01	RHSLLPWFLPGFFPR	Consensus (comb.lib./smm/nn)	2.30	1.1923	No	Positive	0.0806	Negative	0.15
HLA-DRB1^∗^03 : 01	KLSFIECDCRKKRVR	Consensus (smm/nn/sturniolo)	2.40	1.1250	No	Negative	-0.3371	Positive	0.29

## Data Availability

The data used to support the findings of this study are available from the corresponding author upon request.

## Supplementary Materials

Supplementary Materials Table 1. Specific linear B-cell epitopes of *T. gondii* Tyrosine-rich Oocyst Wall Protein predicted by the BCPREDS web server (Threshold 75%). Table 2. Specific linear B-cell epitopes of *T. gondii* Tyrosine-rich Oocyst Wall Protein predicted by the ABCpred web server (Threshold 0.75%). Table 3. Specific linear B-cell epitopes of *T. gondii* Tyrosine-rich Oocyst Wall Protein predicted by the SVMTriP web server.
