# Supplementary material for: Toxoplasma gondii Tyrosine-Rich Oocyst Wall Protein: A Closer Look through an In Silico Prism
Source: Biomed Res Int. 2021 Oct 14;2021:1315618. doi: 10.1155/2021/1315618 (PMC8531782; doi:10.1155/2021/1315618)
Supplement: Supplementary Materials — Table 1. Specific linear B-cell epitopes of T. gondii Tyrosine-rich Oocyst Wall Protein predicted by the BCPREDS web server (Threshold 75%). Table 2. Specific linear B-cell epitopes of T. gondii Tyrosine-rich Oocyst Wall Protein predicted by the ABCpred web server (Threshold 0.75%). Table 3. Specific linear B-cell epitopes of T. gondii Tyrosine-rich Oocyst Wall Protein predicted by the SVMTriP web server. [file 1315618.f1.docx]

**Table 1.** Specific linear B-cell epitopes of ***T. gondii* Tyrosine-rich Oocyst Wall Protein** predicted by the BCPREDS web server (Threshold 75%)

| **Position** | **Epitope** | **Score** |
| --- | --- | --- |
| 219 | PGYGFGGFGPGFGV | 1 |
| 71 | EEAAEPDEDKKDDS | 1 |
| 15 | AVTTRTVAQETEEA | 0.986 |
| 35 | EKVAASSNLTPDNA | 0.98 |
| 107 | KDDKKQGNDEHSSQ | 0.947 |
| 87 | TNNEDEQKGDDDAK | 0.916 |
| 196 | PGYGYGYPGYGYGY | 0.852 |
| 50 | AGAPQNEVAATEKL | 0.803 |
| 176 | PRLSGRQRLLGLGN | 0.728 |

**Table 2.** Specific linear B-cell epitopes of ***T. gondii* Tyrosine-rich Oocyst Wall Protein** predicted by the ABCpred web server (Threshold 0.75%)

| **Position** | **Epitope** | **Score** |
| --- | --- | --- |
| 112 | QGNDEHSSQKLSFI | 0.94 |
| 180 | GRQRLLGLGNLFGG | 0.89 |
| 172 | STMKPRLSGRQRLL | 0.89 |
| 80 | KKDDSEATNNEDEQ | 0.86 |
| 118 | SSQKLSFIECDCRK | 0.85 |
| 37 | VAASSNLTPDNALA | 0.84 |
| 65 | DEKGSGEEAAEPDE | 0.83 |
| 54 | QNEVAATEKLTDEK | 0.81 |
| 167 | QESEGSTMKPRLSG | 0.80 |
| 186 | GLGNLFGGYYPGYG | 0.79 |
| 95 | GDDDAKDHADEQKD | 0.77 |
| 47 | NALAGAPQNEVAAT | 0.76 |
| 148 | KEAFRHSLLPWFLP | 0.76 |

**Table 3.** Specific linear B-cell epitopes of ***T. gondii* Tyrosine-rich Oocyst Wall Protein** predicted by the SVMTriP web server

| **Rank** | **Position** | **Epitope** | **Score** | **Recommended** |
| --- | --- | --- | --- | --- |
| 1 | 5-18 | IKILLLLGLLAVTT | 1.000 | 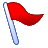 |
| 2 | 142-155 | SCADLVKEAFRHSL | 0.521 |  |
| 3 | 119-132 | SQKLSFIECDCRKK | 0.391 |  |
| 4 | 28-41 | AKLTSDSEKVAASS | 0.333 |  |
| 5 | 55-68 | NEVAATEKLTDEKG | 0.300 |  |
| 6 | 174-187 | MKPRLSGRQRLLGL | 0.219 |  |
| 7 | 97-110 | DDAKDHADEQKDDK | 0.216 |  |
| 8 | 75-88 | EPDEDKKDDSEATN | 0.215 |  |
| 9 | 212-225 | GYPGYGYPGYGFGG | 0.215 |  |
| 10 | 195-208 | YPGYGYGYPGYGYG | 0.215 |  |
